# Are drivers recurring or ephemeral? observations from serial mapping of persistent atrial fibrillation

**DOI:** 10.1093/europace/euae269

**Published:** 2024-10-17

**Authors:** Bram Hunt, Eugene Kwan, Eric Paccione, Benjamin Orkild, Kyoichiro Yazaki, Jake Bergquist, Jiawei Dong, Robert S MacLeod, Derek J Dosdall, Ravi Ranjan

**Affiliations:** Department of Biomedical Engineering, 36 S. Wasatch Drive, SMBB 3100, University of Utah, Salt Lake City, UT 84112, USA; Nora Eccles Harrison Cardiovascular Research and Training Institute, 95 S 2000 E, Bldg. 500, University of Utah, Salt Lake City, UT 84112, USA; Division of Cardiovascular Medicine, Department of Internal Medicine, 30 North Mario Capecchi Dr, 3rd Floor North, University of Utah, Salt Lake City, UT 84112, USA; Department of Biomedical Engineering, 36 S. Wasatch Drive, SMBB 3100, University of Utah, Salt Lake City, UT 84112, USA; Nora Eccles Harrison Cardiovascular Research and Training Institute, 95 S 2000 E, Bldg. 500, University of Utah, Salt Lake City, UT 84112, USA; Division of Cardiovascular Medicine, Department of Internal Medicine, 30 North Mario Capecchi Dr, 3rd Floor North, University of Utah, Salt Lake City, UT 84112, USA; Department of Biomedical Engineering, 36 S. Wasatch Drive, SMBB 3100, University of Utah, Salt Lake City, UT 84112, USA; Nora Eccles Harrison Cardiovascular Research and Training Institute, 95 S 2000 E, Bldg. 500, University of Utah, Salt Lake City, UT 84112, USA; Division of Cardiovascular Medicine, Department of Internal Medicine, 30 North Mario Capecchi Dr, 3rd Floor North, University of Utah, Salt Lake City, UT 84112, USA; Department of Biomedical Engineering, 36 S. Wasatch Drive, SMBB 3100, University of Utah, Salt Lake City, UT 84112, USA; Nora Eccles Harrison Cardiovascular Research and Training Institute, 95 S 2000 E, Bldg. 500, University of Utah, Salt Lake City, UT 84112, USA; Division of Cardiovascular Medicine, Department of Internal Medicine, 30 North Mario Capecchi Dr, 3rd Floor North, University of Utah, Salt Lake City, UT 84112, USA; Nora Eccles Harrison Cardiovascular Research and Training Institute, 95 S 2000 E, Bldg. 500, University of Utah, Salt Lake City, UT 84112, USA; Division of Cardiovascular Medicine, Department of Internal Medicine, 30 North Mario Capecchi Dr, 3rd Floor North, University of Utah, Salt Lake City, UT 84112, USA; Department of Biomedical Engineering, 36 S. Wasatch Drive, SMBB 3100, University of Utah, Salt Lake City, UT 84112, USA; Nora Eccles Harrison Cardiovascular Research and Training Institute, 95 S 2000 E, Bldg. 500, University of Utah, Salt Lake City, UT 84112, USA; Division of Cardiovascular Medicine, Department of Internal Medicine, 30 North Mario Capecchi Dr, 3rd Floor North, University of Utah, Salt Lake City, UT 84112, USA; Department of Biomedical Engineering, 36 S. Wasatch Drive, SMBB 3100, University of Utah, Salt Lake City, UT 84112, USA; Nora Eccles Harrison Cardiovascular Research and Training Institute, 95 S 2000 E, Bldg. 500, University of Utah, Salt Lake City, UT 84112, USA; Division of Cardiovascular Medicine, Department of Internal Medicine, 30 North Mario Capecchi Dr, 3rd Floor North, University of Utah, Salt Lake City, UT 84112, USA; Department of Biomedical Engineering, 36 S. Wasatch Drive, SMBB 3100, University of Utah, Salt Lake City, UT 84112, USA; Nora Eccles Harrison Cardiovascular Research and Training Institute, 95 S 2000 E, Bldg. 500, University of Utah, Salt Lake City, UT 84112, USA; Scientific Computing and Imaging Institute, University of Utah, Salt Lake City, UT, USA; Department of Biomedical Engineering, 36 S. Wasatch Drive, SMBB 3100, University of Utah, Salt Lake City, UT 84112, USA; Nora Eccles Harrison Cardiovascular Research and Training Institute, 95 S 2000 E, Bldg. 500, University of Utah, Salt Lake City, UT 84112, USA; Division of Cardiovascular Medicine, Department of Internal Medicine, 30 North Mario Capecchi Dr, 3rd Floor North, University of Utah, Salt Lake City, UT 84112, USA; Division of Cardiothoracic Surgery, Department of Surgery, University of Utah, Salt Lake City, UT, USA; Department of Biomedical Engineering, 36 S. Wasatch Drive, SMBB 3100, University of Utah, Salt Lake City, UT 84112, USA; Nora Eccles Harrison Cardiovascular Research and Training Institute, 95 S 2000 E, Bldg. 500, University of Utah, Salt Lake City, UT 84112, USA; Division of Cardiovascular Medicine, Department of Internal Medicine, 30 North Mario Capecchi Dr, 3rd Floor North, University of Utah, Salt Lake City, UT 84112, USA

**Keywords:** Atrial fibrillation, Mapping, Rotors, Focal impulse, Driver recurrence

## Abstract

**Aims:**

Rotational re-entries and ectopic foci, or ‘drivers’, are proposed mechanisms for persistent atrial fibrillation (persAF), but driver-based interventions have had mixed success in clinical trials. Selective targeting of drivers with multi-month stability may improve these interventions, but no prior work has investigated whether drivers can be stable on such a long timescale.

**Objective:**

We hypothesized that drivers could recur even several months after initial observation.

**Methods and results:**

We performed serial electrophysiology studies on paced canines (*n* = 18, 27–35 kg) at 1−, 3−, and 6 months post-initiation of continual persAF. Using a high-density 64-electrode catheter, we captured endocardial electrograms in the left atrium (LA) and right atrium (RA) to determine the presence of drivers at each major anatomical site. We defined drivers that were repeatedly observed across consecutive studies to be recurrent. The mean probability that any driver would recur was 66% (LA: 73%, RA: 41%). We also found evidence of ‘multi-recurring’ drivers, i.e. those seen in all three studies. Multi-recurring drivers constituted 53% of initially observed drivers with at least one found in 92% of animals, and we found more multi-recurring drivers per animal than predicted by random chance (2.6 ± 1.5 vs. 1.2 ± 1.1, *P* < 0.001). Driver sites showed more enhancement than non-drivers during late gadolinium enhancement-magnetic resonance imaging (*P* = 0.04), but we observed no relationship between enhancement and driver recurrence type.

**Conclusion:**

We observed recurring drivers over a 6-month period at fixed locations, confirming our hypothesis. We also found drivers to be associated with fibrosis, implying a structural basis.

## Graphical Abstract

**Graphical Abstract:**
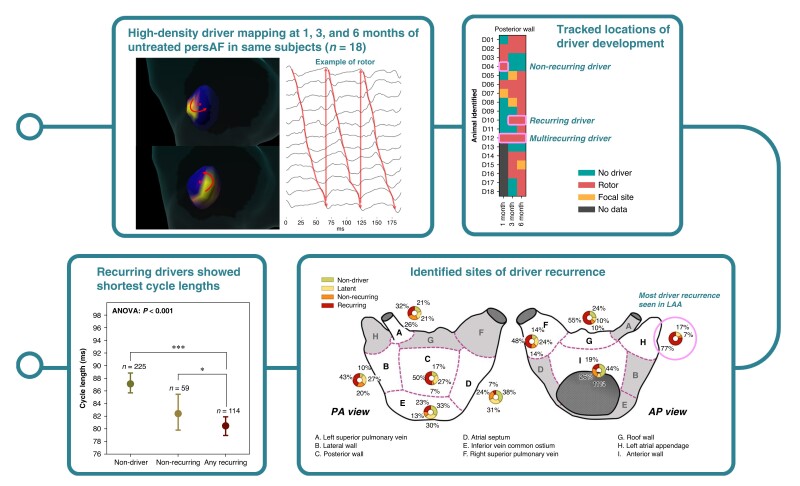

**What’s New?**

- Serial endocardial mapping studies in a paced canine model of persistent atrial fibrillation revealed a subset of drivers to recur in the same locations over several months.
- Sites of recurring drivers predominate in the left atrium and exhibit shorter cycle lengths compared with non-recurring drivers, and 66% of all drivers recurred at the next mapping study. Recurring drivers were most prevalent in the left atrial appendage, where 92% of drivers were recurring.
- Sites with drivers showed more enhancement than sites with non-drivers during late gadolinium enhancement-magnetic resonance imaging, suggesting structural relevance to driver activity, but recurring drivers did not show more enhancement than non-recurring drivers.

## Introduction

Recent evidence has linked persistent atrial fibrillation (persAF) to the existence of spatially stable disruptive phenomena known as drivers.^1,2^ These drivers include pinwheel re-entries, termed *rotors*, and focal activations. However, efforts to utilize drivers as targets for catheter ablation have been mixed in terms of efficacy.^3–6^ Explanations for these results may include variation in operator technique, lack of standardized driver treatment strategies, or low mechanistic importance of drivers. Some studies have shown low temporal stability of purported driver mechanisms,^7–9^ which may be evidence of a poor substrate basis for these mechanisms. However, contemporary examinations of persAF are generally limited to a single study to evaluate rotational and focal drivers before treatment.^10^ Given that the structural remodelling thought to be responsible for drivers develops on a months-long rather than minutes-long basis, drivers would be expected to be found at the same sites even on a months-long basis. However, we have seen no such examinations in the literature, and because contrary results would undermine arguments for the treatment of driver sites, an investigation is merited. Since serial clinical studies with extensive mapping are infeasible clinically, a large animal model of persAF is best suited to perform this study.

A large number of mapping methodologies have been used to identify and localize drivers,^11^ including contact mapping, optical mapping, multi-electrode mapping in the epicardium, moderate density basket mapping, and electrocardiographic imaging (ECGI). Each methodology has advantages and disadvantages, but they all generally suffer from some combination of poor spatial resolution, incomplete atrial coverage, unknown generalizability of simulated cardiac potentials, and inappropriateness for serial survival studies. Advancements in catheter technology have allowed for high-density mapping with endocardial basket catheters. These catheters offer excellent spatial resolution for driver identification, broad substrate coverage, and real cardiac potentials. They have also been validated as a method of driver identification.^12^

In this study, we sought to determine the long-term time-dependent changes in driver recurrence and location. We hypothesized drivers to be consistently found in the same locations across multiple chronologically separate electrophysiological studies. To do this, we utilized high-density catheters to capture endocardial unipolar signals in a paced canine model of persAF over a period of 6 months. Evidence of stability across several months would better justify the use of drivers as targets in ablation. We also sought to determine the linkage of structural remodelling to both drivers and temporally stable drivers and provide evidence for the ability of high-density mapping to detect drivers.

## Methods

For all studies, we adhered to the Guide for the Care and Use of Laboratory Animals.^13^ The Institutional Animal Care and Use Committee at the University of Utah approved the protocol.

### Paced canine model

Mixed breed mongrel hounds were used (*n* = 18, 27–35 kg, 1–2 yrs.). We implanted neurostimulators with screw-in bipolar pacing leads in the right atrium (RA) at either the right atrial appendage or the lateral wall. Initially, we paced at 50 Hz for 1 s every other second. Every 1–2 weeks afterwards, we ceased pacing and electrocardiogram recordings to determine whether atrial fibrillation (AF) was persistent. For this study, we defined persAF as the maintenance of AF for longer than 20 min following cessation of external stimulation. After an animal was determined to be in persAF, we lengthened the pacing interval to 1 s of pacing per minute to ensure re-initiation of AF if the animal converted to sinus rhythm. Most animals entered persAF 3 weeks after we started the pacing protocol. We continued to verify that animals were sustaining AF with weekly electrocardiogram recordings. Spontaneous termination of AF was not observed for any animal while recording the local EGMs during the EP studies, and the animals maintained a mean AF duration of 6 months. While no significant differences in the number of drivers were expected between sexes, both male (*n* = 6) and female (*n* = 12) canines were used to evaluate the impact of sex.

### Mapping studies

We performed serial electrophysiological studies on the canines at 1, 3, and 6 months after AF was sustained. We stopped all pacing and thereafter used the Rhythmia Mapping System and 64-electrode high-density Orion catheter (Boston Scientific) to create high-density endocardial maps of sustained AF.^14^ All geometries were created from the Rhythmia Mapping System’s internal impedance mapping software in coordination with magnetic tracking and under intracardiac echo (ICE) guidance to ensure that the entire atrium was covered.

After the geometry was created, the catheter was moved around the atria to record AF activity. For this procedure, we moved the catheter to a stable site in the atrium, ensured good contact, and then held the catheter at that location to acquire 4 min of electrograms. The operators determined contact by manoeuvring or inserting the catheter to maximize voltage in each site of interest and verified the arrival of the catheter to the desired anatomical location by observing the catheter in real time in ICE, and its position in the 3D atrial geometry was created using fluoroscopy and ICE. After recording, we moved the catheter to a new site, and we repeated this process until the entire left atrium (LA) and RA was covered, which required between 26 and 34 individual recordings (104–136 min of electrograms) per study. Pacing was resumed after the completion of each study.

Animals were fasted for a minimum of 12 h before any surgical procedure and then were sedated using propofol (5–8 mg/kg intravenous) and intubated for operation. Anesthesia was then applied through inspired, vaporized isoflurane (1.5–4%). We used 8.5 and 9 Fr sheaths (Abbott) for gaining femoral vein access and 5 Fr sheaths in the femoral artery to monitor blood pressure. No heparin was administered. This was done to minimize post-procedure bleeding. For transseptal access, we used NRG transseptal needles (Baylis), and access was gained with ICEcardiogram and fluoroscopy guidance. All animals presented in AF at the start of the EP procedures and sustained AF for the entire duration of the mapping study.

### Magnetic resonance imaging

For each animal, we performed late gadolinium enhancement-magnetic resonance imaging (LGE-MRI) 1 week before the 6-month electrophysiology study after cardioverting the animals to sinus rhythm. Briefly, LGE-MRI was acquired 20 min after injecting Gd-BOPTA (0.15 mmol/kg; Bracco Diagnostic Inc, Princeton, NJ) using a respiratory-navigated, electrocardiogram-triggered, inversion-recovery-prepared 3D-gradient echo sequence with a spatial resolution of 1.25 × 1.25 × 2.5 mm, TR/TE = 3.1/1.4 ms, flip angle = 14°, and an inversion time (TI) of 230 to 330 ms.^10^We used Seg3D (Scientific Computing and Imaging Institute, University of Utah, Salt Lake City, UT) to manually segment each LGE-MRI scan into atrial wall, blood pool, and regions of atrial enhancement. The segmented atrial walls were then non-rigidly registered to the 6-month atrial geometries created in Rhythmia, which allowed direct comparison between the manually segmented areas of enhancement and mapping data.

Then, for each site measurement that we performed in our 6-month electrophysiology study, we determined what fraction of the surface area nearest to the catheter (<5 mm) had LGE-MRI enhancement. We compared this fraction between sites with and without drivers.

### Driver identification

For each study performed, we examined the unipolar atrial AF electrograms to find either rotational or focal mechanisms that could be driving the AF by dividing the atrium into 15 major anatomical sites and inspecting relevant recordings from each site. For each anatomical site, we inspected the 2 s of recording with the highest dominant frequency, using common filters described elsewhere.^15^ With these 2 s of electrograms, we eliminated QRS-T artefacts with average beat subtraction and removed powerline noise with a 60 Hz notch filter.^16,17^ We took the first temporal derivative of these cleaned signals to create movies of activation sequences. In cases where more than one recording was performed at a single site, we looked at the recording with the higher dominant frequency. See Supplementary material online, *Appendix S1* for example visualizations of these movies and examples of identified drivers. For each anatomical site, a reviewer (BH) inspected these movies and annotated the locations of rotational or focal activation patterns. To be classified as a ‘driver,’ our criteria required sites to exhibit ≥3 consecutive rotations or focal activation patterns.

### Recurrence measures

**Figure 1:**
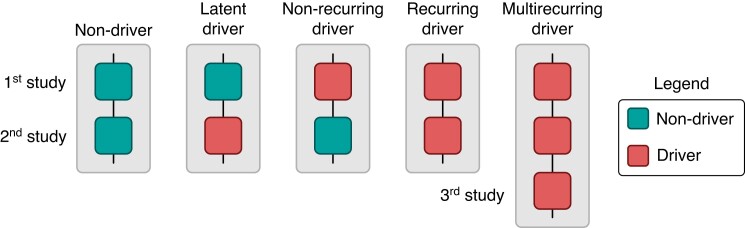
Visualization of recurrence measures. For repeated measures at the same anatomical site across two sequential studies, we examined the presence of drivers and categorized them into four classes: recurring drivers, non-recurring drivers, latent drivers, and non-drivers. Additionally, if a site exhibited a driver across all three studies, we classified it as having a multi-recurring driver.

To evaluate cross-study recurrence of drivers, we compared changes in the presence of a driver between the 1- and the 3-month studies or between the 3- and the 6-month studies for each major anatomical site and animal. In these comparisons, we classified any given site by its *future* state (e.g. a currently observed driver that will not be observed at the next study is a *non-recurring driver*). We outlined four classes: recurring drivers, non-recurring drivers, latent drivers (i.e. unobserved in the current study but appears in a later study), and non-drivers. Examples are shown in *Figure 1*. As an additional consideration, we made a special analysis of drivers found in all three studies, which we term *multi-recurring* drivers. We examined the number and distribution of all types of driver recurrence to evaluate anatomical site differences.

To some degree, drivers will recur in subsequent studies with a simple, random distribution of drivers throughout the atrium. In such cases, recurring drivers would be a fluke rather than having any real basis in the substrate. To discount this possibility, we simulated the random allocation of drivers throughout the atria to determine how many recurring and multi-recurring drivers would be seen based on mere chance. See Supplementary material online, *Appendix S2* for the details of this process.

We also quantified the strength of associations between cycle length and driver recurrence type. For any given anatomical site, we computed cycle length as the reciprocal of the maximum dominant frequency for the entire 4-min AF electrogram. For any driver at a given site, we calculated the cycle length of that driver as being that of the site where it was observed.

To determine whether recurring and multi-recurring drivers would be excluded by conventional PVI, we examined each study performed and examined whether the exclusion of drivers in the pulmonary veins would have left at least one recurring driver remaining.

### Statistical analysis

Averages are reported as mean ± SD. For comparing continuous variables between more than two groups, we used ANOVA to determine significance. We performed two group comparisons with *t*-tests, paired *t*-tests, Wilcoxon signed rank tests, and Mann–Whitney *U* tests as appropriate. For categorical variables, we used Fisher’s exact test and McNemar’s test as appropriate. Inter-rater and intra-rater analyses were performed with Cohen’s kappa.

## Results

### Drivers and anatomical sites

*Table 1* shows the numbers of identified rotors, focal points, and non-drivers throughout the major anatomical sites of the LA and RA. The rates of prevalence of a driver at the average anatomical site were 56% for the LA and 25% for the RA. On an individual site basis, we found that the LA appendage had the highest probability of containing a driver (83%). The LA lateral wall (65%) and LA roof (65%) had the next highest probabilities. For the RA, the appendage had the highest fraction of drivers per observation (45%). In a blinded review of driver classification repeatability, we found Cohen’s kappa for the intra-rater analysis of the reviewer (BH) to be 0.68 with 84.6% agreement (*n* = 26 sites). Cohen’s kappa for the inter-rater analysis (BH vs. BO) was 0.77 with 88.5% agreement (*n* = 26 sites).

**Table 1:** Distribution of driver sites in the atria across all canine studies (*n* = 18 canines)

Site	Percentage of Sites (*n*)		
	Rotor	Focal	Non-Driver
LA Anterior	23% (11)	13% (6)	64% (30)
LA Appendage	67% (33)	16% (8)	16% (8)
LA Lateral	61% (30)	4% (2)	35% (17)
LA LIPV	42% (20)	0% (0)	58% (28)
LA LSPV	26% (9)	23% (8)	51% (18)
LA Posterior	57% (28)	8% (4)	35% (17)
LA Roof	49% (24)	16% (8)	35% (17)
LA RSPV	55% (26)	9% (4)	36% (17)
LA Septum	34% (16)	0% (0)	66% (31)
RA Appendage	43% (19)	2% (1)	55% (24)
RA IVC	20% (9)	11% (5)	68% (30)
RA Lateral	9% (4)	2% (1)	89% (40)
RA Posterior	18% (7)	3% (1)	80% (32)
RA Septum	19% (8)	7% (3)	74% (31)
RA SVC	10% (4)	5% (2)	86% (36)

Each row shows the breakdown of classifications for a given site, i.e. the percentage of such sites classified as a rotational driver, a focal driver, or a non-driver (planar or non-reentrant wavefronts). For each electrophysiological study, the number of distinct measurements ranged between 30 and 37 in the LA and between 31 and 34 in the RA. Across all studies, the total number of sites examined in the LA and RA was 420 and 257, respectively.

### Changes in driver number

**Figure 2:**
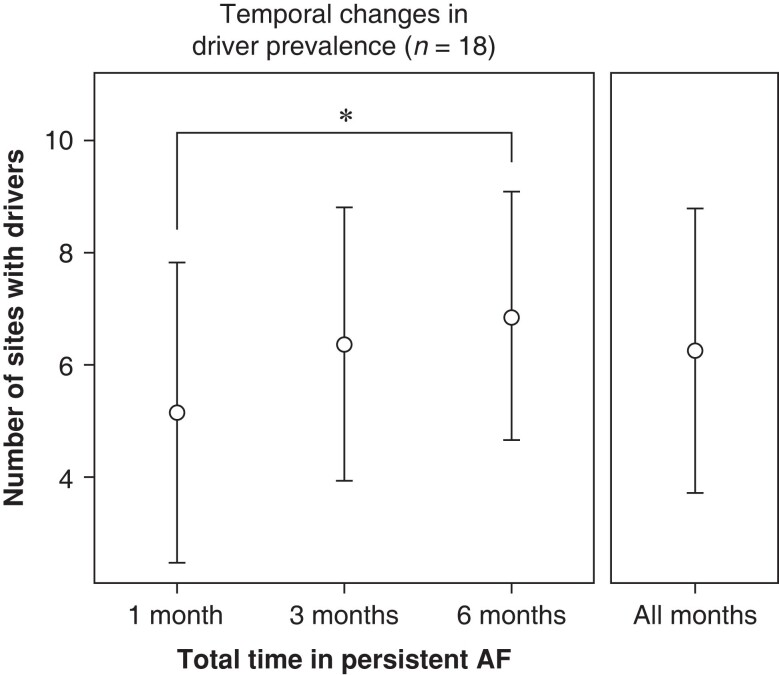
Number of drivers found per study date in 18 canines. The number of drivers increased with time in AF between the 1- and the 6-month studies (*P* = 0.04, *t*-test). Driver totals for 1-, 3-, and 6-months were 5.2 ± 2.8, 6.4 ± 2.5, and 6.9 ± 2.3, respectively. The mean number of drivers for any given animal and study was 6.3 ± 2.5.

Across all studies, the mean number of identified drivers per study was 6.3 ± 2.5. *Figure 2* shows changes in this total over the time course of the paced persAF. We observed an increase in driver number across all studies (*P* = 0.048, ANOVA), and we found that the total number of drivers increased significantly between the 1-month and the 6-month studies (*P* = 0.043). Mean driver totals for 1−, 3−, and 6-months were 5.2 ± 2.8, 6.4 ± 2.5, and 6.9 ± 2.3, respectively. The mean numbers of drivers found in the LA and RA were 4.9 ± 1.9 and 1.4 ± 1.1, respectively, with the LA having a significantly greater number (*P* < 0.0001, *t*-test). Of the identified drivers, 82% (248/301) were rotors and 18% (52/301) were focal. The left superior pulmonary vein and left atrial appendage had the highest numbers of focal drivers with prevalence rates of 23% (8/34) and 16% (8/49), respectively. The number of drivers per study was 5.1 ± 2.4 for males (*n* = 6) and 6.8 ± 2.4 for females (*n* = 12), and we found no significant differences between the sexes (*P* = 0.11, *t*-test).

### Temporal changes in driver sites

**Figure 3:**
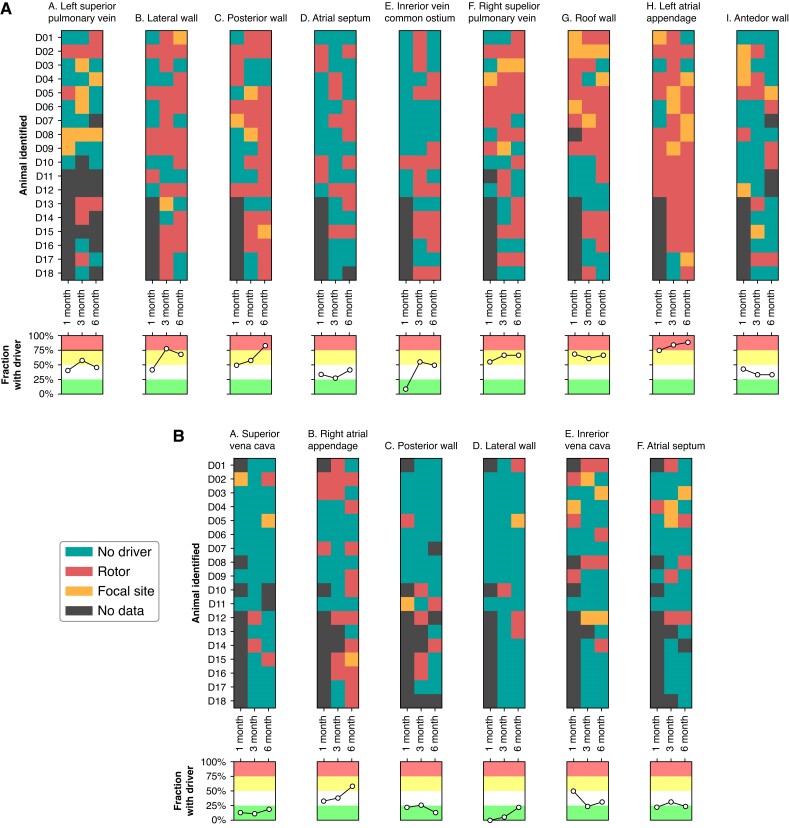
Identified drivers stratified by anatomical site, measured animal (*n* = 18), and electrophysiology study date. Each block corresponds to an anatomical site, with colour indicating the existence and type of driving mechanisms. The line plots show the fraction of the above blocks with drivers at the given study date. For some sites, we could not classify the site due to insufficient data quality, early termination of non-reinducable AF, or animal unavailability for study at the desired date. (*A*) identified drivers in the LA. (*B*) identified drivers in the RA.

*Figure 3* tracks individual drivers across all three studies for each animal and anatomical site in the LA and RA. Whereas the average driver number increased with time, an evaluation on an individual animal basis showed low consistency in driver presence from site to site. However, the final electrophysiology study revealed a driver in the LA appendage in all but two animals (89%) and a driver in the posterior wall in all but three animals (83%).

### Driver recurrence

**Figure 4:**
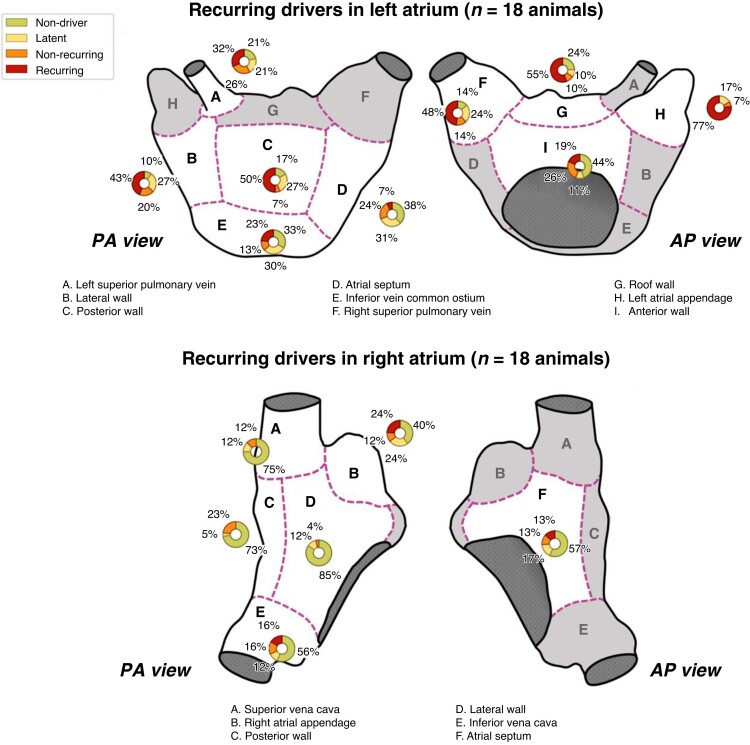
Observed drivers by recurrence type and anatomical sites. We defined recurring drivers as sites where a driver was observed in two consecutive studies. Sites where greater than 50% of drivers were recurring were the roof and the appendage. Recurring drivers constituted 73% and 41% of all drivers in the LA and RA, respectively.

In *Figure 4*, we show the frequencies of anatomical sites with recurring, non-recurring, and latent drivers as well as those lacking a driver (non-drivers). In general, 66% (114/174) of observed drivers were recurring. The diagnostic odds ratio for correctly predicting that a site would possess a driver in the future was 3.8 ± 0.2 (sensitivity: 66%, specificity: 66%). Whereas latent drivers were greater in number than non-recurring drivers, the time of persAF between studies was non-preferential for either crossover type (*P* = 0.14, McNemar’s). The probability of a driver recurring was 72% in the LA and 41% in the RA, with the LA being significantly more likely to have drivers recur than the RA (*P* < 0.01, Fisher’s exact). In terms of recurring drivers per observed drivers, the sites with the highest rates of driver recurrence were the LA appendage (92%), LA posterior wall (88%), LA roof (84%), and the RSPV (78%). We found no difference in recurrence rates between focal and rotational drivers (*P* = 0.42, Fisher’s exact).

### Multi-recurring drivers

Of all drivers observed in the initial study, 53% (31/59) were multi-recurring (i.e. observed for both following studies), and 92% (11/12) of animals had at least one multi-recurring driver. For drivers observed in the first two studies, we found that 76% (31/41) of these drivers were also observed in the 6-month study. As seen in *Table 2*, the LA contained 97% (30/31) of all multi-recurring drivers. The site that showed multi-recurring drivers the most frequently was the LA appendage, with 67% (8/12) of subjects possessing an LA multi-recurring driver. As for whether multi-recurring drivers were seen more frequently than expected by random chance, our simulation results showed that the observed number of multi-recurring drivers per animal exceeded that predicted by random chance (2.6 ± 1.5 vs. 1.2 ± 1.0, *P* < 0.001, Mann–Whitney *U* test).

**Table 2:** Locations, number, and percentage of total for multi-recurring drivers

Site	Probability of having multi-recurring driver
LA Appendage	67% (8/12)
LA Roof	55% (6/11)
LA RSPV	36% (4/11)
LA Lateral	33% (4/12)
LA Posterior	33% (4/12)
LA LSPV	25% (2/8)
LA Anterior	11% (1/9)
RA Appendage	11% (1/9)
LA LIPV	8% (1/12)
LA Septum	0% (0/12)
RA IVC	0% (0/8)
RA Lateral	0% (0/9)
RA Posterior	0% (0/8)
RA Septum	0% (0/8)
RA SVC	0% (0/7)

All these drivers but one were found in the LA. The most common site for the multi-recurring drivers was the LA appendage.

### Markers of recurring and multi-recurring drivers

**Figure 5:**
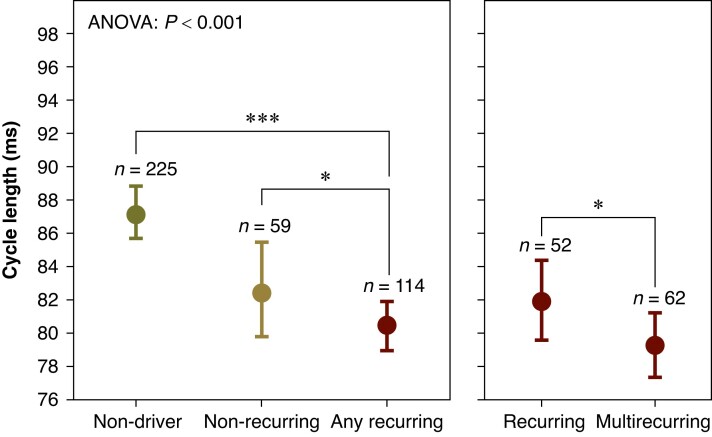
Distributions of cycle length as a function of driver recurrence type. We observed a relationship between types with respect to cycle length for both recurrence and multi-recurrence (*P* < 0.01 and *P* < 0.0001 respectively, ANOVA). We also observed sites with recurring and multi-recurring drivers to have significantly shorter cycle lengths than other sites, even for non-recurring and singly recurring drivers. This observation supports cycle length as a marker for both driver recurrence and multi-recurrence.

*Figure 5* shows observed cycle lengths for each type of driver recurrence. We found that there was a significant overall difference between recurrence types (*P* < 0.001, ANOVA), and that recurring drivers exhibited shorter cycle lengths than any other type of site, including non-recurring drivers (recurring vs. non-driver: *P* < 0.0001; recurring vs. latent: *P* < 0.01; recurring vs. non-recurring: *P* = 0.02, *t*-test). We also found that multi-recurring drivers exhibited shorter cycle lengths than singly recurring drivers (*P* < 0.05, *t*-test).

**Figure 6:**
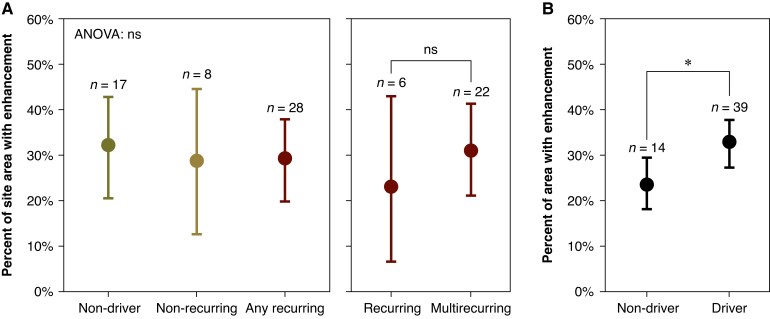
Relationship between driver recurrence type and LGE-MRI enhancement. For each animal (*n* = 6), enhanced regions of LGE-MRI were manually segmented as fibrotic. We excluded animals without all three studies, and we excluded two animals that lacked high-quality MRI. For each site, we calculated what percentage of area within 5 mm of the measurement centre was fibrotic. There was no relationship between driver recurrence type and enhancement (*P* = 0.74, ANOVA). We found that sites with a driver had higher levels of enhancement than sites without drivers (*P* = 0.04, Wilcoxon signed rank test). (*A*) recurrence type and enhanced area. (*B*) existence of driver and enhanced area.

More broadly, we observed the sites of drivers to have shorter cycle lengths than the sites of non-drivers (82 ± 12 ms vs. 90 ± 13 ms, *P* < 0.0001, Mann–Whitney *U* test). No significant difference in cycle length was observed between rotors and focal drivers (82 ± 11 ms vs. 83 ± 13 ms, *P* = 0.58, Mann–Whitney *U* test). We found that LGE-MRI enhancement was significantly related to the existence of a driver (*P* = 0.04, Wilcoxon signed rank test), as shown in *Figure 6*. We found no relationship between fibrosis fraction and driver multi-recurrence (*P* = 0.74, ANOVA).

### Effects of PVI from a temporally informed perspective

For recurring and multi-recurring drivers, respectively, only 24% (27/114) and 22.6% (7/31) were present in the pulmonary veins. For our 36 studies of animals with recurring drivers, PVI would have left at least one recurring driver untreated in 28 studies. For multi-recurring drivers, PVI would exclude them in 10 of the 12 animals.

## Discussion

Many contemporary investigations of persAF recurrence have considered the multiple driver hypothesis, i.e. that rotational mechanisms and non-pulmonary vein ectopies are the primary causal factors of persAF after PVI. Efforts to ablate these drivers, although initially promising, have met with mixed success in large-scale trials.^1–3^ Our results add crucial pieces to this puzzle by evaluating the time stability of drivers over multiple studies separated by several months. Our findings show a complex picture of driver recurrence, with many drivers being transient. However, we also find the existence of what we term *recurring* and *multi-recurring* drivers, potentially offering merit to more selective driver ablation strategies that target them.

### Drivers and anatomical sites

Ignoring temporal changes in driver presence, we found the distribution of driving mechanisms in the atrium to be heterogeneous with respect to the atrium and specific anatomical sites. Most prior work focused on LA drivers and LA ablation for AF, and our results support this approach across a number of developmental time points in the same animals. The rate of probability of locating a driver at any given anatomical site in the LA was >50%, which would allow even random ablations to have some chance of targeting a rotor core or focal site. For example, roofline lesions between the pulmonary veins have been attempted with mixed success at terminating persAF, which may be explained by a high innate potential for driver formation in the LA roof enabling even random ablation to target a driver. Our work also supports LA appendage isolation on a mechanism termination basis, as nearly three quarters of our LA appendages had at least one driver. The free wall had a greater proportion of drivers than average, but no previous studies have shown this nor have prior treatment strategies focused on free wall ablation beyond PVI. In terms of RA treatment, our results bolster the validity of ablation strategies targeting the RA appendage, given its demonstrated propensity for driver development. The degree to which this targeting is explained by the increased contact area of the high-density catheter in the appendage is unknown. Potentially, a reduction in persAF recurrence by isolation in both atria may be better attributed to the reduction in fibrillatory area.^18,19^ In the same vein, this study’s findings support patient-specific rather than generalized driver ablation strategies because no particular site had 100% driver occurrence rates. Finally, we observed 19% (3/16) of animals to have a superior vena cava driver at 6 months, and this proportion lies within the range of other studies that have indicted the SVC as a frequent source of non-pulmonary AF.^20–22^

### Changes in driver number

Past studies have shown differences in driver number between patients with paroxysmal and persistent AF, but they have not determined whether a driver development timeline exists, or to what extent, in persAF. To our knowledge, ours is the first study to show a long-term driver development timeline for persAF. Additionally, we now have strong evidence that the continued structural remodelling of the heart during persAF leads directly to increases in the number of drivers within that substrate. Large-scale clinical trials have shown that maintenance of sinus rhythm reduces patient mortality and improves the quality of life,^23^ and our work provides an explanation for this finding by showing evidence of significant, maladaptive remodelling at play when AF is allowed to continue.

### Temporal changes in driver sites

PVI is a standard for persAF ablative treatment, and our results justify PVI even for paced persAF.^24^ All but two animals had a driver in one of the pulmonary veins by 6 months of fibrillation. In contrast to varieties of AF originating from ectopic beats in the pulmonary veins, this finding may indicate the existence of pathogenic pathways for arrhythmogenic substrate in the veins that can be triggered by AF. We likewise found common development of drivers in the LA appendage, roof, and lateral wall. A standardized driver ablation design would likely incorporate all these sites.

### The left atrial appendage

We observed rapid development and near-universality of drivers in the LA appendage. Previous work has identified the LA appendage as a common site for driver development, with up to 27% of patients exhibiting appendage firing.^25^ This finding starkly contrasts with our finding where nearly all animals developed a driver in the LAA by 6 months. Our finding may be explained by differences in mapping technology (decapolar vs. 64-electrode mapping catheters), length of patient population in AF, or differences between human and canine electrophysiology.

Left atrial appendage isolation (LAAI) has been proposed and tested as treatment for persAF, with LAAI and PVI improving over PVI for freedom from AF at 12 months (56% vs. 28%).^26,27^ The great number of drivers in the LA appendages in our study supports this treatment from a theoretical basis, but prior evidence has shown that LAAI increases the risk of embolic stroke,^28^ complicating the use of LAAI as a general strategy. If direct ablation of rotor cores and ectopies is ineffective in treatments for persAF such that isolating methods are preferred, then future work must carefully balance the risks and benefits resulting from such an isolation procedure. Not all voltage-mapping modalities enable precise measurement of the LA appendage. From a methodological perspective, our findings bolster the importance of choosing modalities that can capture electrograms in the LA appendage.

Finally, the canine LA appendage is approximately the same size as a human LA appendage and thus constitutes a larger proportion of the LA due to the smaller gross size of the canine heart. If persAF driver distribution is related to the relative size of anatomical components, our results may overestimate the impact of the appendage.

### Drivers and remodelling

Cardiac fibrosis is classically concurrent with arrhythmia. This fibrosis becomes a potential substrate for re-entry by introducing fibrotic block and conduction slowing throughout the heart. Atrial fibrosis, therefore, has long been a target of investigation in the search for AF mechanisms, and the advent of late gadolinium enhancement as a marker for structural remodelling has allowed for *in vivo* investigation. In this study, we used LGE-MRI to assess average anatomical site fibrosis. The effectiveness of using LGE-MRI as a surrogate for ablative scarring is well established, as shown through many prior studies, and there is evidence that the method is appropriate for the coarse detection of fibrosis. Previously, we histologically observed the development of fibrosis in our model, providing additional justification for our use of LGE-MRI to detect fibrosis in this study.^10^

We have provided evidence of a linkage between regions of higher fibrosis and the existence of drivers at said regions, which falls in line with other studies that have likewise associated drivers to fibrosis with both LGE-MRI^29–32^ and simulated methods.^33,34^ This contrasts with Chrispin et al (2016) and Sohns et al (2017), who observed no correlation between rotors and LGE-MRI fibrosis.^35,36^ This may be attributed to the use of differing driver identification methodologies, as they used FIRM mapping and ECGi, respectively, which is highlighted by the fact that with a similar number of patients in the two studies (9 and 10, respectively), they identified 18 vs. 410 rotors. We used a high-density mapping system to record the local electrograms, which was distinctly different for both of these studies. Additionally, these studies examined cohorts with a mix of fibrosis and ablation scar, whereas we strictly looked at pre-ablation fibrosis with no dense scar areas, and it is possible that drivers are associated with fibrosis only within a narrow range of fibrotic density before dense scar is formed, potentially altering the relationship between drivers and atrial fibrosis. Overall, our findings support the hypothesis that drivers have some degree of real basis in structure. Our study does not elucidate the exact nature of that basis, however, as we examined only average fibrosis across regions.

Given that no other studies have examined the reoccurrence of drivers across multiple months, no evidence exists as to whether reoccurring drivers have a different relationship with fibrosis from other drivers. In our study, we observed a non-significant increase in average fibrosis between regions with multi-recurring and singly recurring drivers, although we were limited by our small sample size of usable LGE-MRI scans with multi-recurring drivers. A larger sample is needed to have more definitive conclusions.

### Driver recurrence

Our data are uniquely able to show recurrence of drivers over multiple, repeated studies in the same heart. In a stagnant, non-changing atrial environment, driving sites and non-driving sites would not vary from study to study. However, the great proportion of mechanisms changing from driver to non-driver and vice versa shows persAF to remain highly dynamic and defy generalization.

We do not have easy answers to driver ablation from our serially mapped perspective of persAF. It is natural to assume that if a driver were *recurring*, then it would have an underlying and diseased substrate that is destroyable by ablation. If all drivers were recurring, then it would be easy to propose continuing contemporary efforts to ablate driving mechanisms. However, although identified drivers were typically recurring, a similar proportion of sites possessed latent drivers. These latent drivers may be responsible for later recurrence if they develop into active drivers by a mechanism unobservable by voltage mapping (cellular or otherwise). In that case, so long as these sites are unable to be identified, driver ablation may largely be doomed to fail except in cases of collateral ablation of these sites, but the probability of such collateral ablation is unknown. When combining the existence of latent drivers with the large proportion of drivers that fail to recur, we see a poor prognosis for effective driver ablation, unless operators are able to predict recurrence patterns. If recurrence patterns were indeed predictable, these latent sites may be good targets for treatment.

### Cycle length as a predictor of recurrence

Motivated to find evidence that recurrence type might be predictable, we quantified the relationship between cycle length and recurrence type. As expected, we found recurring drivers to have the shortest cycle length of any recurrence type. This finding is significant because it suggests (I) sites with recurring drivers possess some manner of functional basis that distinguishes them from other sites, and (II) these recurring driver sites may be predictable in their development. Future work should further investigate functional markers for recurring drivers.

### Multi-recurring drivers

For nearly all animals, we observed at least one site with a driving mechanism present in all three electrophysiology studies. We propose these multi-recurring drivers to be sites with the arrhythmogenic substrate remaining stable over several months, which would coincide with the expected time of development of structural- rather than functional-based changes to the myocardium. Thus, these drivers may reflect structure in their maintenance. However, we primarily found a functional marker, cycle length, correlated with driver multi-recurrence, which may reflect site-specific long-term functional remodelling that reinforces itself to become more stably arrhythmogenic with time.

Regardless, considering their repeated reappearances in multiple studies, multi-recurring drivers may be a first choice for ablation in a hierarchical approach to driver treatment, but future studies are needed for confirmation.

## Conclusions

To the best of our knowledge, this is the first study to evaluate multi-month changes in driver recurrence and location. We confirmed our initial hypothesis, finding a subset of identified drivers in early studies to recur in later studies. The LA roof, appendage, and RSPV were the most likely sites for driver recurrence, and generally, the LA had more recurring drivers than the RA. Finally, we showed evidence for the existence of multi-recurring drivers. These multi-recurring drivers demonstrated faster cycle lengths than singly recurring or non-recurring drivers, and their presence across several months may indicate an entrenched arrhythmogenic substrate.

## Limitations

We captured data for between two and four min for each anatomical site in our electrophysiology studies, but we evaluated only the 2-s windows of the highest activation frequency for each site. Relatedly, our standards for driver identification were strict (≥3 consecutive rotations or ectopic beats). These two factors may have resulted in a higher Type II error rate. This higher rate may have reduced the proportions of recurring and multi-recurring drivers. We also made no investigation of meandering rotors.

As for our choice of model, canine models are considered the ‘gold standard’ for non-clinical studies of persAF, including structural and functional components of the disease. Structurally, the atrial sizes of our paced canines (60–100 ccs. per chamber) are similar to human atrial sizes, and we have previously observed paced canines to develop substantial fibrotic remodelling as validated by histology.^10^ However, we did not observe significant areas of dense scar that are typical of patient cohorts. Functionally, canines have higher resting heart rates and can sustain even higher rates, potentially enabling a greater number of rotational re-entries via a reduction in wavelength. However, we largely found the number of drivers to be within the range of prior work on human AF^2^. Finally, the pathogenesis of clinically observed persAF is dependent on patient-specific conditions and may be present for extensive lengths of time. Our canine model may develop in a more predictable manner due to a common pathogenetic cause (i.e. rapid pacing of the RA) and is limited to only 6 months of persAF, and as such, may not reflect the diversity of conditions present in human persAF.

## Supplementary Material

euae269_Supplementary_Data

## Data Availability

The data underlying this article are available upon reasonable request.
