# Supplementary material for: Are drivers recurring or ephemeral? observations from serial mapping of persistent atrial fibrillation
Source: Europace. 2024 Oct 17;26(11):euae269. doi: 10.1093/europace/euae269 (PMC11542584; doi:10.1093/europace/euae269)
Supplement: euae269_Supplementary_Data [file euae269_supplementary_data.docx]

**Supplementary Appendix 1 | Activation Mapping of Atrial Fibrillation.**

We created a software suite for identifying drivers at specific anatomical locations. Examples of how the Orion catheter was visualized is shown in **Figure A1**. This graphical user interface (GUI) allowed for real-time geometry navigation, replay speed changes, filtering, and electrode inspection. **Figures A2 and A3** show use of the software to identify a rotor and focal driver respectively. A site was classified as possessing a driver if the reviewer observed 1) three consecutive rotational reentries such that continuous conduction was observed in the same local area (i.e., 1080° of rotation), or 2) three consecutive focal activations without interruption in the same local area. This was done while scanning over the 2 seconds of highest activation frequency for a given site.


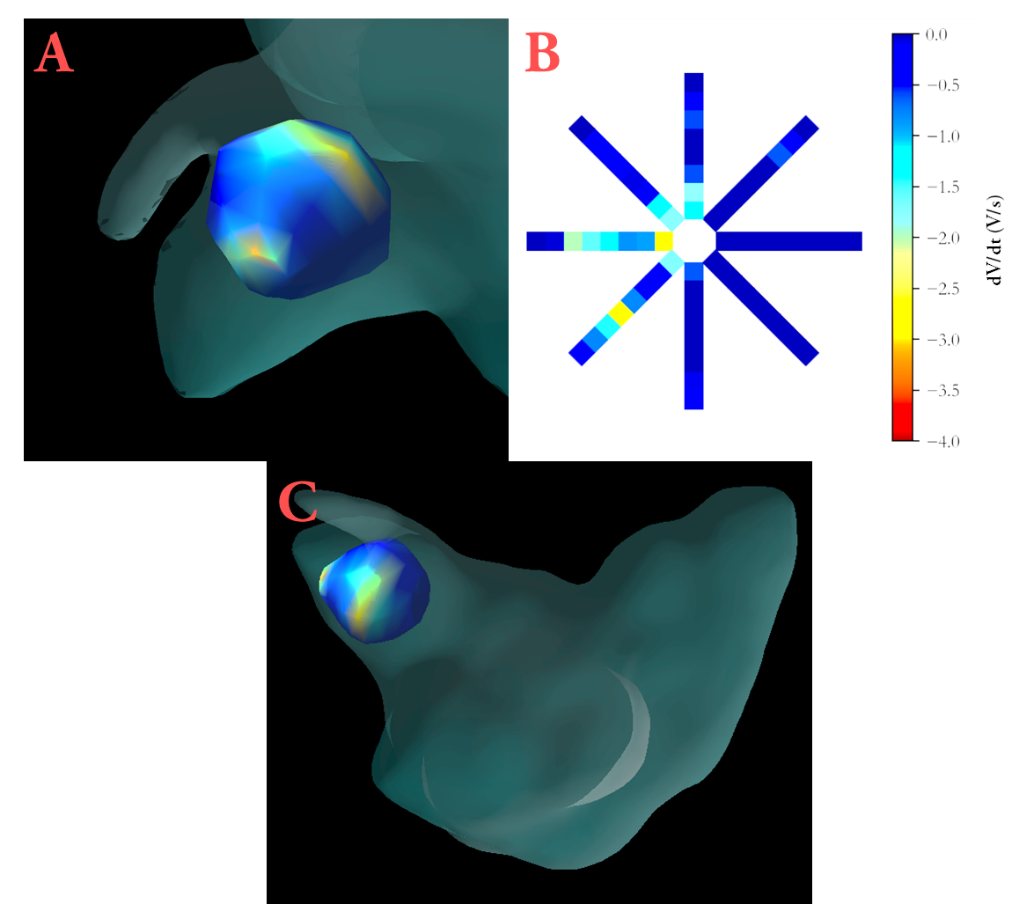


**Figure A1. Activation maps during atrial fibrillation in an example canine LA.** We identified rotors and focal activations using video playback of dV/dt at individual anatomical sites. A) Example of 64-electrode Orion catheter placed in the left atrial appendage with electrodes colored according to derivative value with interpolation. B) Splayed view of electrodes used in visualizing veins. C) Global view of catheter position.


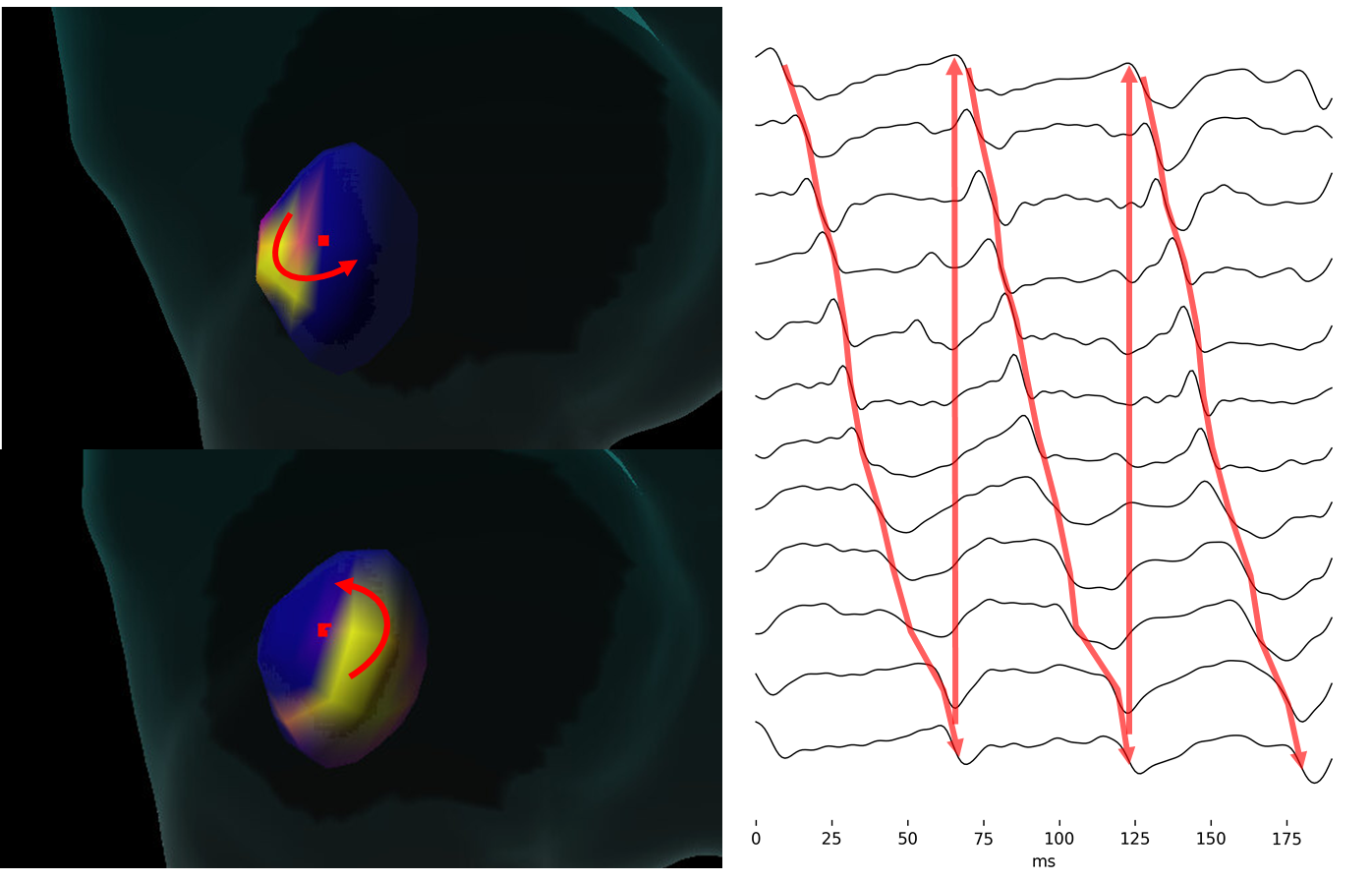


**Figure A2. Mapping and identification of a rotational driver with electrogram readout.** An example rotational driver with clockwise conduction in the left atrial posterior wall (left) and an electrogram readout of the underlying electrograms (right). Arrows indicate direction of conduction, and the red square indicates the center of the rotor. Regions of high dV/dt are colored in blue and regions of low dV/dt are colored in yellow.


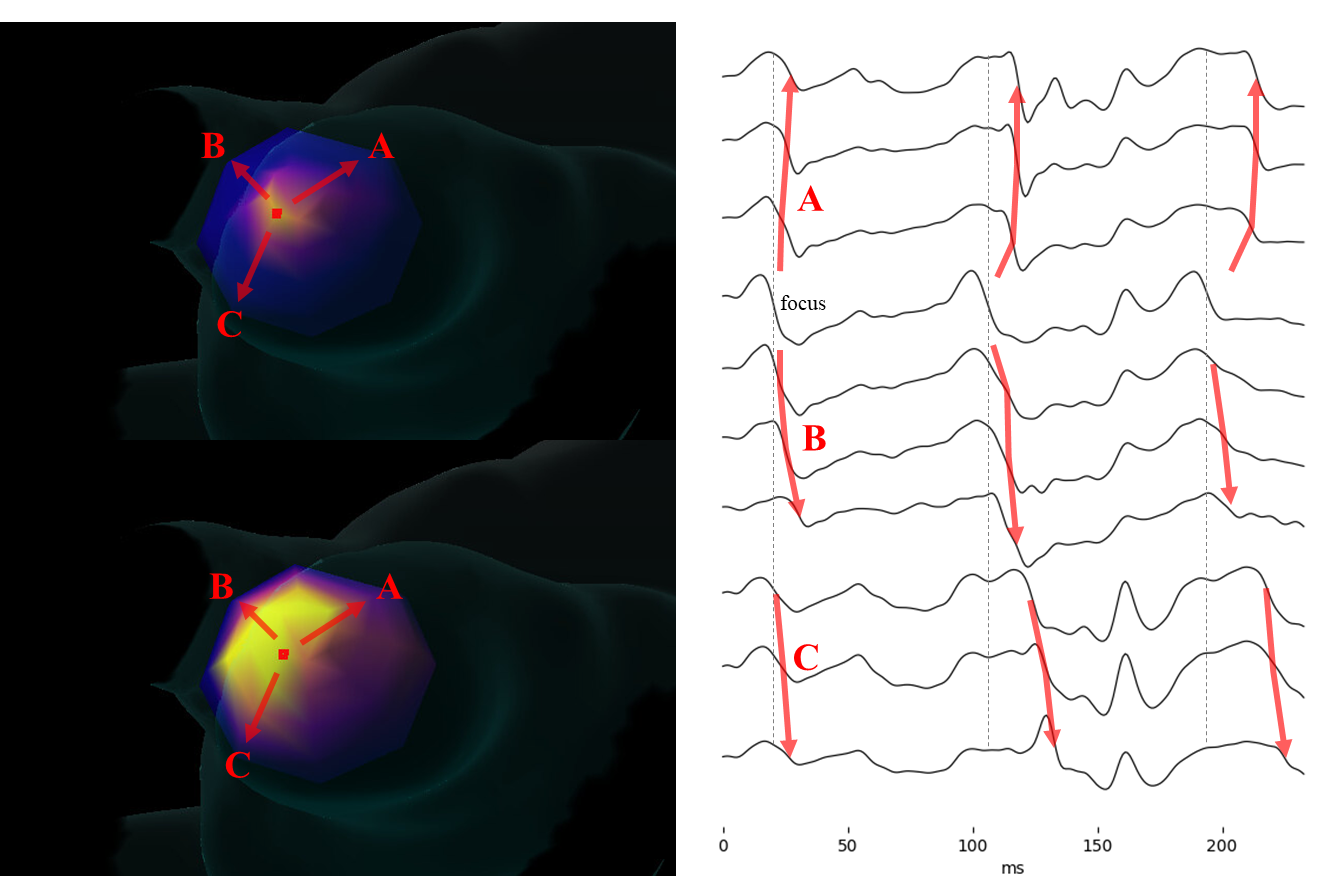


**Figure A3. Mapping and identification of a focal driver with electrogram readout.** An example focal driver in the left atrial appendage (left) and an electrogram readout of the underlying electrograms (right). Arrows indicate direction of conduction, the red square indicates the origin of the focus, and the dashed lines are isochrones at the activation times of the focus. Regions of high dV/dt are colored in blue and regions of low dV/dt are colored in yellow.

**Supplementary Appendix 2 | Determination of Random Recurrence.**

To determine the random probability of observing recurring drivers given our methods, we used a Monte Carlo method to simulate driver allotment throughout the atria. In these simulations, we placed a set number of drivers randomly throughout both atria according to the distributions we observed in our serial mapping studies.

For example, at the 1-month mapping study, we observed an average of 4.2±1.7 drivers in the left atrium and 1.3±1.1 drivers in the right atrium across nine and six anatomical sites respectively. To simulate a 1-month study in the left atrium, we randomly sampled our observed distribution to acquire a number of drivers to randomly places across the nine measured sites in the left atrium. We repeated this protocol 10,000 times to simulate driver distributions in the left and right atria at 1-, 3-, and 6-months using their respective observed distributions in our real data. This created 10,000 simulated animals that underwent our serial mapping protocol with drivers at random sites.


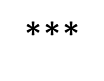

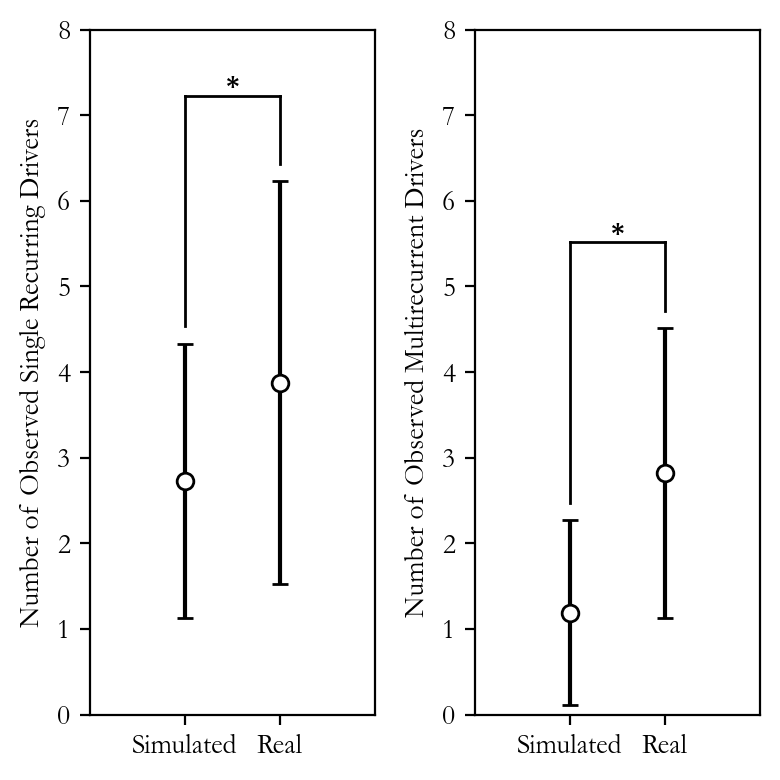
From these 10,000 simulated animals, we found an average number of recurring drivers that would occur by random chance. We found the simulated number of both single and multirecurring drivers to be significantly fewer than those observed *in vivo*. **Figure A4** shows these distributions.

**Figure 4. Distributions of the number of recurring drivers in both real and simulated data.** The real number of observed recurring drivers was greater than that observed in the simulated data (3.9±2.4 vs. 2.7±1.6, p=0.03, Mann-Whitney U test), and the real number of multirecurring drivers was similarly greater than in the simulated data (2.8±1.7 vs. 1.2±1.1, p<0.001, Mann-Whitney U test).
